# Functional Visual Acuity of Early Presbyopia

**DOI:** 10.1371/journal.pone.0151094

**Published:** 2016-03-09

**Authors:** Yusaku Katada, Kazuno Negishi, Kazuhiro Watanabe, Yuta Shigeno, Megumi Saiki, Hidemasa Torii, Minako Kaido, Kazuo Tsubota

**Affiliations:** Department of Ophthalmology, Keio University School of Medicine, Shinanomachi, Shinjuku-ku, Japan; Seoul St. Mary's Hosptial, REPUBLIC OF KOREA

## Abstract

**Purpose:**

To evaluate visual function in patients with early presbyopia using the functional visual acuity (FVA) test.

**Methods:**

This study included 27 eyes of 27 healthy older volunteers (mean age, 44.1 ± 2.6 years) and 14 eyes of 14 healthy young volunteers (mean age, 28.4±4.8 years). The distance-corrected visual acuity (DCVA), distance-corrected near VA (DCNVA), subjective amplitude of accommodation (AA), and distance and near pupillary diameters were measured. The distance FVA and distance-corrected near FVA (DCNFVA) were measured using the FVA Measurement System. The standard Schirmer test and standard tear break-up time measurement also were performed.

**Results:**

The logarithm of the minimum angle of resolution (logMAR) DCVA was better than 0 in all subjects. The percentages of subjects with logMAR DCNVA below 0 was significantly lower in the presbyopia group than in the young group. The DCNFVA in the presbyopia group was significantly (*P* < 0.001) poorer than the DCNVA in that group. Significant linear negative correlations were seen between the DCNVA and AA (r = -0.507, *P* < 0.001) and the DCNFVA and AA (r = -0.681, *P* < 0.001) in the older subjects. Stepwise regression analysis showed that only the AA was a significant factor predictive of the DCNFVA in the presbyopia group. Tear function parameters were not adopted in the regression model.

**Conclusions:**

Measurement of the DCNFVA can detect decreased AA in early presbyopia better than measurement of the conventional near VA. The DCNFVA is a good index for early presbyopia.

## Introduction

Presbyopia is age-related loss of accommodation that results in near visual disturbance. Early presbyopia is characterized by a variety of symptoms including decreased reading performance[1–3] and asthenopia or headache following close work even without decreased near visual acuity (VA).[4] The loss of accommodation is the main etiology of decreased near vision in normal aging subjects. However, age-related changes in the near visual performance can be related to several factors other than loss of accommodation such as contrast sensitivity,[5–8] pupillary size,[9–12] aberrations,[13–19] and light scatter.[13,20,21]Conventional near VA testing and measurement of the accommodation amplitude (AA) cannot always reflect the near visual performance in aging subjects.

Conventional visual acuity testing has been traditionally accepted for assessing the visual function, but conventional testing may have limitations when assessing the quality of vision. To simulate functional vision of daily life, functional VA (FVA) was first defined by Goto et al[22]. In brief it is measured as the average of the visual acuities measured during a certain time frame, so it reflects daily vision more efficiently than measuring visual acuity at 1 specific point. The FVA test has been reported to be an important method of defining detailed visual function that cannot be detected by conventional VA testing.[22–27] The FVA first was developed to detect impaired visual function in daily activities in dry eye syndrome, and it has recently been applied to assessments of visual function in various conditions including soft contact lens wear,[28] eye drop instillation,[29] laser in situ keratomileusis,[26] posterior capsule opacification,[30] early cataract,[31] and astigmatism.[32] Those reports have demonstrated the usefulness of FVA for detecting decreased visual function in eyes with good VA.

We assessed visual function in subjects with early presbyopia using the FVA test.

## Methods

### Subjects

Twenty-seven eyes of 27 healthy subjects ranging in age from 40 to 49 years in the presbyopia group, and 14 eyes of 14 subjects ranging in age from 20 to 34 years in the young group were enrolled in this prospective study from the general population at the Department of Ophthalmology, Keio University School of Medicine. The participants had no ophthalmic disease other than refractive error. Only one eye of each subject with the lower refractive error was included. Subjects were excluded when the subjective refraction (spherical equivalent) was less than -4.0 diopters (D) or greater than +4.0 D. The institutional review board of Keio University School of Medicine approved this study, and all subjects provided written informed consent. The study adhered to the tenets of the Declaration of Helsinki.

### Conventional VA, Amplitude of Accommodation, and Pupillary Diameter Measurements

**Fig 1:**
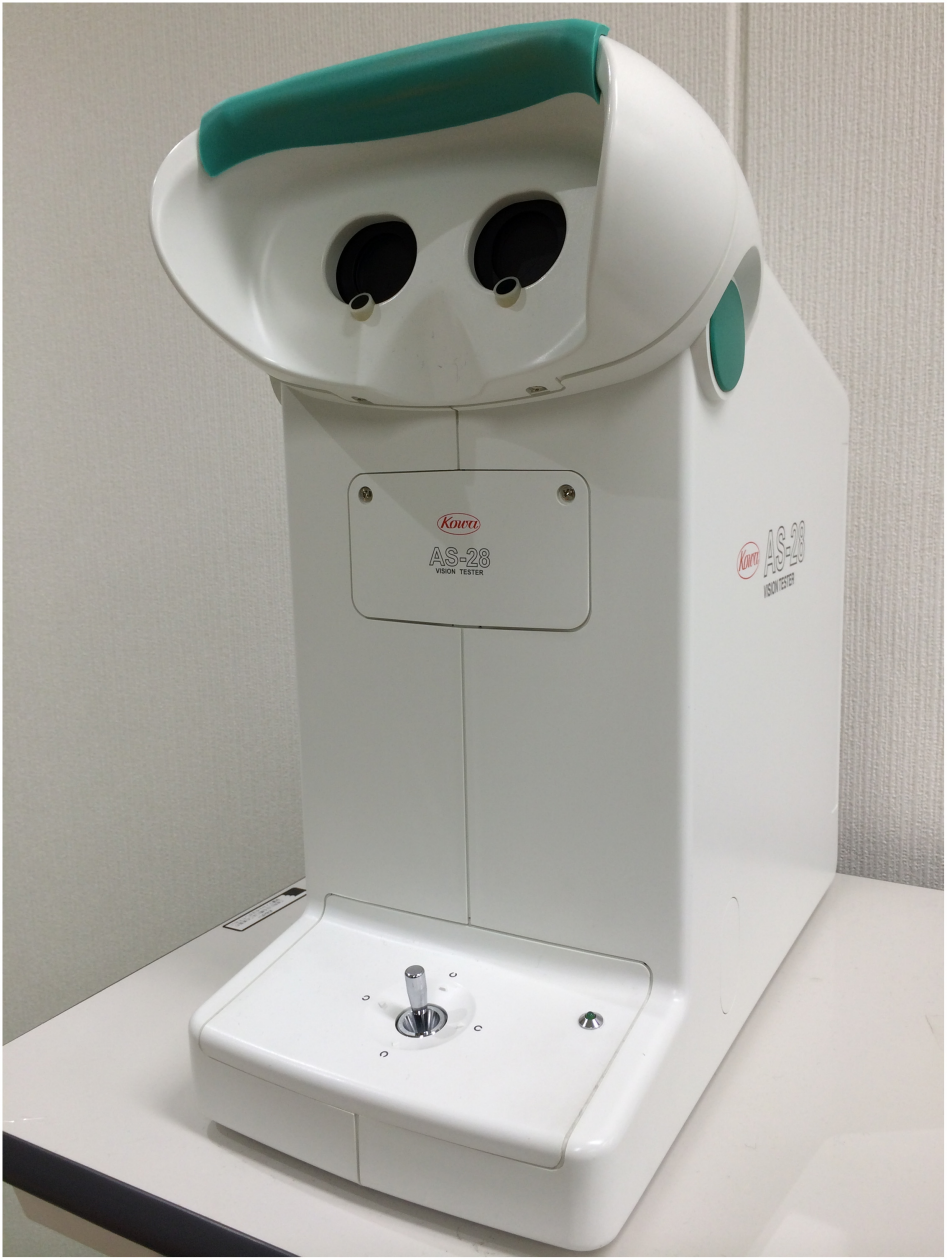
The Functional Visual Acuity Measurement Device, the Kowa AS-28.

All subjects underwent an examination that included measurement of the distance-corrected VA (DCVA) and distance-corrected near VA (DCNVA) using the FVA Measurement System, the AS-28 (Kowa, Aichi, Japan) (Fig 1). The subjective amplitude of accommodation (AA) was measured using the D’Acomo dioptric accommodator (World Optical, Kyoto, Japan). The distance and near pupillary diameters were measured using the FP-10000 pupillometer (TMI, Saitama, Japan).

### FVA Measurement

All subjects underwent measurements of the distance functional visual acuity (DFVA) and distance corrected near functional VA (DCNFVA).

**Fig 2:**
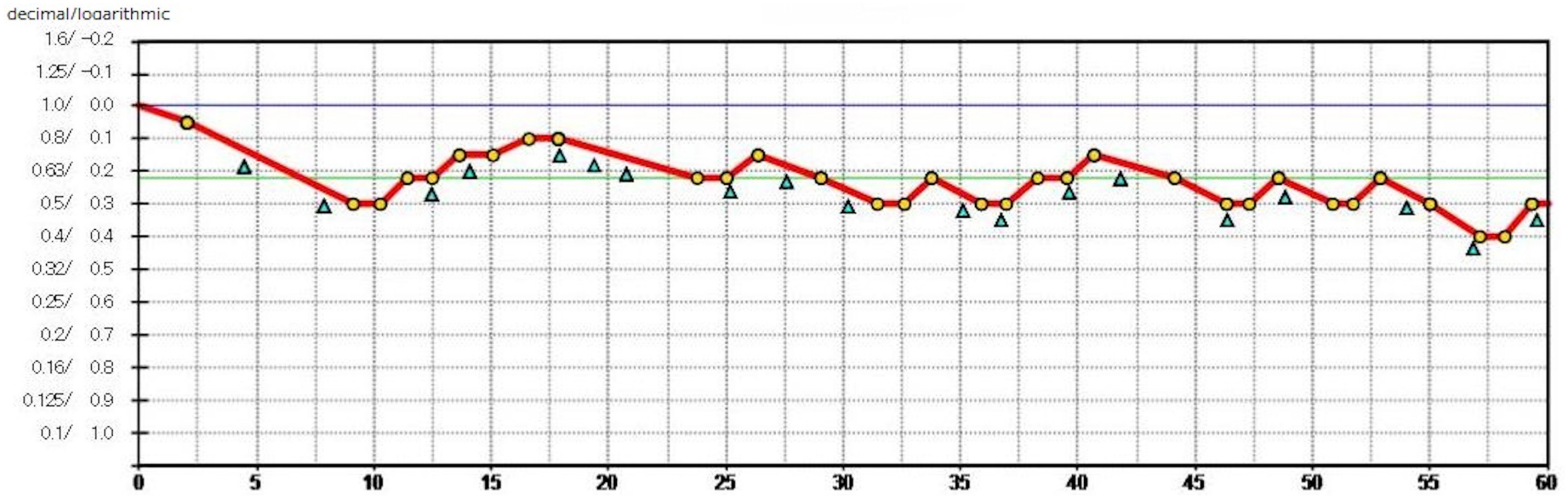
A representative printout of typical functional visual acuity (VA) testing. The blue line denotes the Landolt corrected VA. The red line shows the time-wise changes in the VA during testing. The green line denotes the mean logarithm of the minimum angle of resolution (logMAR) over 60 seconds, defined as the functional VA. The yellow dots show the number of correct responses; the blue triangles indicate spontaneous blinks.

The FVA Measurement System was used to examine the time-wise changes in continuous VA over 60 seconds. The Landolt optotypes are presented in the device, and their sizes change depending on the correctness of the responses. The measurement starts with the best-corrected Landolt VA, which is the baseline VA for each individual. The Landolt optotypes decrease in size automatically with correct answers; when the responses are incorrect, larger optotypes are presented automatically. When there is no response within the set display times, the answer is considered an error and the optotype enlarges automatically. Subjects delineate the orientation of the automatically presented Landolt rings using a joystick. The system can measure VA levels from 30/20 to 20/200. The presentation time of an optotype was adjusted to 2 seconds, and the optotypes changed automatically even if the subjects missed responding within 2 seconds. The VA during 60 seconds was recorded in this study. The testing was performed with the subjects blinking spontaneously, and the continuous VA changes were plotted (Fig 2). The FVA measurement included the FVA, maximal and minimal VAs, and average response time. The FVA was defined as the average of all VA values measured over time because this average may reflect the daily vision more accurately than the VA measured at a specific time point. The DFVA was measured with the best correction for distance; the DCNFVA was measured with -3.00 D added to the best distance correction, which was equivalent to the mean DCNVA measured continuously for 60 seconds.

### Tear Function Evaluation

All subjects underwent the standard Schirmer’s test without topical anesthesia and the standard tear break-up time (BUT) measurement. Standardized strips of filter paper (Showa Yakuhin, Tokyo, Japan) were placed in the lateral canthus away from the cornea and left in place for 5 minutes with the subjects’ eyes closed. The BUT measurement was performed after instillation of fluorescein sodium in the conjunctival sac with test paper (Showa Yakuhin). The interval between the last complete blink and the appearance of the first black corneal spot in the stained tear film was measured three times, and the mean value was calculated.

### Statistical Analysis

Comparisons of the DCVA, DCNVA, DFVA, and DCNFVA were performed using the paired Student’s *t*-test. The Mann-Whitney *U*-test was performed to compare the DCVA, DCNVA, DFVA, and DCNFVA between older and younger subjects. Pearson’s correlation analysis was used to study the correlations among age, conventional VA, FVA, AA, and tear functions parameters. *P* < 0.05 was considered statistically significant. Stepwise regression analysis was performed to investigate the relation between the DCNFVA and age, AA, near pupillary diameter, Schirmer’s test score, and BUT score. All statistical analyses were performed with SPSS software version 12.0J for Windows (IBM, Armonk, NY).

## Results

### Conventional VA, FVA, AA, and Pupillary Diameter Measurement

The Table 1 shows the clinical data of the subjects. The logarithm of the minimum angle of resolution (logMAR) CDVA was better than 0 in all subjects. The percentages of subjects with a logMAR DCNVA under 0 was significantly lower in the presbyopia group than in the young group. The DCNFVA in the presbyopia group was significantly (*P* < 0.001) poorer than the DCNVA in that group.

**Table 1:** Characteristics of the Study Participants.

Parameters	Presbyopia Group (Mean ± SD)	Young Group (Mean ± SD)	*P* Value
Age (years)	44.1 ± 2.6	28.4 ± 4.8	<0.001*
CDVA < 0 (logMAR)	100% (-0.169 ± 0.026)	100% (-0.176 ± 0.000)	-
DCNVA < 0 (logMAR)	48.1% (0.142 ± 0.267)	100% (-0.156 ± 0.051)	<0.001^†^
DFVA (logMAR)	-0.119 ± 0.038	-0.114 ± 0.048	0.740
DCNFVA (logMAR)	0.335 ± 0.334	-0.119 ± 0.041	<0.001*
AA (D)	3.26 ± 1.09	9.19 ± 1.98	<0.001*
Distance pupillary diameter (mm)	3.28 ± 0.615	4.01 ± 0.89	<0.001^‡^
Near pupillary diameter (mm)	2.67 ± 0.435	3.15 ± 0.438	0.002*
Schirmer’s test (mm)	8.7 ± 7.9	16.0 ± 11.7	0.039^‡^
BUT (seconds)	5.6 ± 2.9	7.5 ± 4.7	0.257

SD, standard deviation; logMAR, logarithm of the minimum angle of resolution; DCVA, distance-corrected visual acuity; DCNVA, distance-corrected near visual acuity; DFVA, distance functional visual acuity; DCNFVA, distance-corrected near functional visual acuity; AA, amplitude of accommodation; BUT, tear-film break-up time; D, diopters.

*P < 0.05, Student’s *t*-test test.

^†^*P* < 0.05, chi-square test.

^‡^*P* < 0.05, Mann-Whitney’s *U*-test.

**Fig 3:**
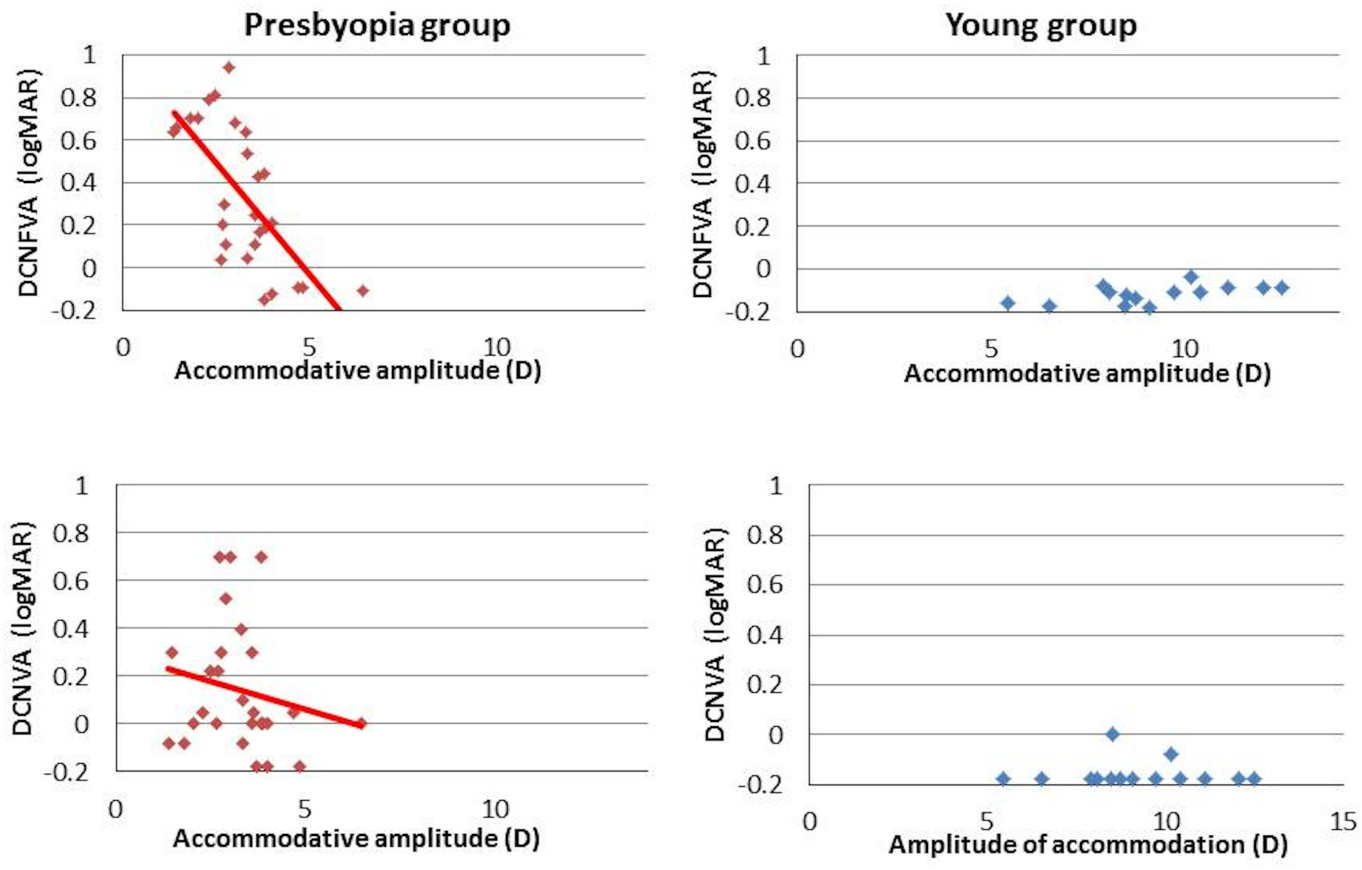
Correlations between the distance-corrected near visual acuity (DCNVA) and amplitude of accommodation (AA), the distance-corrected near functional visual acuity (DCNFVA), and AA in each group. The vertical axis shows the VA and the horizontal axis shows the AA. Significant linear correlations are seen in both of the presbyopia group’s combinations, and the slope of the linear regression between the DCNFVA and AA is steeper than between the DCNVA and AA. D, diopters.

There were significant linear negative correlations between age and AA (r = -0.920, *P* = 0.001), between the DCNVA and AA (r = -0.507, r^2^ = 0.257, *P* < 0.001), and between the DCNFVA and AA (r = -0.681, r^2^ = 0.464, *P* < 0.001) in the presbyopia group (Fig 3). According to regression analysis, the decreases of the DCNVA and DCNFVA for a reduction of 1.00 D of accommodative power were—0.050 ± 0.011 and—0.075 ± 0.013 respectively. The decrease in the DCNFVA was greater than that of the DCNVA.

The distance and near pupillary diameters in the presbyopia group were significantly smaller than those in the young group.

### Tear Function Evaluation

The Schirmer’s test scores in the presbyopia group were significantly lower than those in the young group. There was no significant difference in the BUT score.

### Multiple Regression Analysis

Stepwise regression analysis showed that only the AA was a significant (R^2^ = 0.463, *P* < 0.001) prognostic factor for the DCNFVA in the presbyopia group. No tear function parameters were entered into the regression model. There was no multicollinearity among the variables. The multiple regression equation was as follows: DCNFVA = (-0.209 × AA) + 1.017.

## Discussion

In the current study, we measured the near visual performance in patients with early presbyopia using the FVA test. The results showed that the DCNVA and DCNFVA were correlated significantly with the AA, and the DCNFVA detected decreased near visual performance in early presbyopia better than the DCNVA.

The age-related loss of accommodation is a main etiology of presbyopia. However, various possible factors can degrade the near visual function with aging other than loss of accommodation. For example, with aging, contrast sensitivity decreases,[5]–[8] pupillary size decreases,[10] the density of the crystalline lens increases,[33] and intraocular light scatter and optical aberrations increase,[34],[35] all of which can affect the near visual function.

Older adults have impaired contrast sensitivity under photopic conditions at intermediate and high spatial frequencies.[5],[6],[8] The loss of spatial contrast sensitivity at low luminance levels in older adults is accentuated compared to the loss under photopic conditions including a decline in the high spatial frequency cut-off in older adults compared with that in young adults.[36] Slowing of the visual processing speed with age also has been reported.[37–39] Schefrin et al.[36] reported age-related declines in contrast sensitivity values irrespective of accommodation. Akutsu et al.[2] identified an age-related decline in reading speed, which they suggested is due to age-related losses in visual contrast sensitivity. Teramoto et al.[40] described a strong effect of accommodative power on reading at near but also suggested the effect of decreased retinal illuminance and contrast sensitivity. Those previous studies have shown that it may be insufficient to evaluate the daily near visual performance in older adults based on only the AA or conventional VA testing.

The concept of FVA was first proposed and defined as vision for daily activities in dry eye patients. However, FVA testing has been reported useful for evaluating detailed visual function recently.[23]–[25] Those previous studies found that FVA reflects more sharply reduced visual function than conventional VA testing. For example, Goto et al.[22] reported that the mean FVA decreased significantly in subjects with dry eye with good conventional VA testing. Yamaguchi et al.[31] saw improvements in FVA after cataract surgery in patients with mild cataracts and visual symptomatology despite a good preoperative and postoperative conventional VA. Wakamatsu et al.[30] reported that the mean FVA improved significantly after neodymium-doped yttrium aluminium garnet capsulotomy in parallel with an improvement in the results of conventional VA testing. Watanabe et al.[32] found that FVA testing detected masked visual impairment due to astigmatism more effectively than conventional VA measurement. We also observed that FVA may be able to detect a masked visual impairment due to presbyopia more effectively than conventional VA.

Our results and previous reports suggest that the FVA system is useful for evaluating not only optical disorders but also accommodative dysfunction due to aging. Applications of the FVA measurement system could be expanded to various conditions and unidentified disorders.

It is difficult to diagnose degraded near visual function in subjects with early presbyopia with good conventional VA clinically and quantitatively. In the current study, the DCNFVA detected early presbyopia better than DCNVA measurement, which suggested that the FVA is a good index to identify near visual function and presbyopia.

In the current study, stepwise regression analysis showed that the DCNFVA was correlated only with the AA. As mentioned previously, near visual function in presbyopia is affected by pupillary size [41], but in the current study the near pupillary diameter was not adopted as a prognostic factor for the DCNVA. This may be because the current study did not include subjects 35 to 39 years of age in whom the reduced pupillary size begins to no longer cover the loss of accommodation. It is thought that among the patients with progressed loss of accommodation like the current study, the pupillary diameter does not significantly affect the near visual function.

The FVA is affected by astigmatism and ocular surface disorders.[22],[32] In the current study, astigmatism was corrected at the time of measurement, but neither the Schirmer’s score nor the BUT was adopted for stepwise regression analysis. This may be because in a previous report the effect of dry eye on the FVA was measured with topical anesthesia in order to manifest the dry eye.^22^ In the current study, the measurement was performed without topical anesthesia, so the dry eye was considered to not significantly affect the FVA.

The current study had some limitations. First, the near vision was measured by adding a -3.00-D lens artificially in the FVA Measurement System, which may not reflect precisely the near vision performance in a natural environment. Further studies are needed to clarify the effect of this factor. Second, there was a bias of subject number and gender between the two groups. The current study also investigated the effects of only age, AA, pupillary diameter, and tear function. Future studies on the effect of scattering, contrast sensitivity, and aberration are essential.

In conclusion, the current study showed that DCNFVA can detect decreased AA in early presbyopia better than the conventional near VA measurement. The DCNFVA is a good index for identifying early presbyopia.
